# Anisotropic Friedel oscillations in graphene-like materials: The Dirac point approximation in wave-number dependent quantities revisited

**DOI:** 10.1038/s41598-018-19730-2

**Published:** 2018-02-08

**Authors:** Tohid Farajollahpour, Shirin Khamouei, Shabnam Safari Shateri, Arash Phirouznia

**Affiliations:** 1 Department of Physics, Azarbaijan Shahid Madani University, 53714-161 Tabriz, Iran; 2 Condensed Matter Computational Research Lab, Azarbaijan Shahid Madani University, 53714-161 Tabriz, Iran; 3 Department of Physics, University of Bonab, Bonab, East Azarbaijan Iran

## Abstract

Friedel oscillations of the graphene-like materials are investigated theoretically for low and intermediate Fermi energies. Numerical calculations have been performed within the random phase approximation. It was demonstrated that for intra-valley transitions the contribution of the different Dirac points in the wave-number dependent quantities is determined by the orientation of the wave-number in *k*-space. Therefore, identical contribution of the different Dirac points is not automatically guaranteed by the degeneracy of the Hamiltonian at these points. Meanwhile, it was shown that the contribution of the inter-valley transitions is always anisotropic even when the Dirac points coincide with the Fermi level (*E*_*F*_ = 0). This means that the Dirac point approximation based studies could give the correct physics only at long wave length limit. The anisotropy of the static dielectric function reveals different contribution of the each Dirac point. Additionally, the anisotropic k-space dielectric function results in anisotropic Friedel oscillations in graphene-like materials. Increasing the Rashba interaction strength slightly modifies the Friedel oscillations in this family of materials. Anisotropy of the dielectric function in k-space is the clear manifestation of band anisotropy in the graphene-like systems.

## Introduction

One of the richest and most thriving fields of condensed matter physics is two dimensional structures. Experimental observation of graphene in 2004^1,2^ created a great motivation in scientists to study the intriguing properties in other two-dimensional (2D) allotropes of IV group elements such as silicene, germanene^3^ and recently stanene^4,5^. These new 2D materials and other buckled honeycomb lattice structures predicted in theoretical works^6–9^ and several experimental synthesization have also been performed for realization of these materials^10–13^. These silicon and germanium analogues of graphene with slightly buckled honeycomb geometry have been predicted to have cone-like band energy around the Dirac points where electrons follow the massless Dirac equation near the Fermi level^6–8^. The hybridization of *π* bonds in silicene is not pure and the structure of silicene shows a mixed hybridization. The *π* electrons in silicene are much more active and this leads to a different structure from graphene^14^. Similar to the graphene structure, silicon atoms are arrayed in a hexagonal lattice, with a slight buckling that proved by first principle studies where it has been shown that low buckled silicene is thermally stable^7^. It was also shown that the electronic dispersion of the silicene near *K* points of the first Brillouin zone is linear similar to the behavior of Dirac materials^6,7,15,16^. The spin orbit coupling (SOC) in silicene is stronger than that of graphene which leads to relatively large energy gap at the Dirac points. Strong SOC in silicene makes this monolayer a good candidate for topological insulators and quantum spin Hall effect^8,17–19^. The unique optical and electronic properties of graphene-like systems such as silicene have made these materials a good candidate for plasmonics applications. Meanwhile, plasmonic-based studies have already been performed for graphene, however, the other graphene-like systems are known as highly appealing subjects for this field of condensed matter physics^20–23^.

It has been generally assumed that the degeneracy of the Dirac points provides the identical contribution of these points in the physical quantities. This could be considered as a correct and valid procedure for calculation of the scalar quantities. However, in this case the band anisotropy of the honeycomb structures is completely disregarded. Identical treatment of the Dirac points automatically ignores anisotropy of the band energy. It seems that this anisotropy could be appeared just at high Fermi energies. However, as it was shown in this work, even in the case of low Fermi energies where the Fermi level could match the Dirac points the contribution of inter-valley transitions are completely anisotropic.

Within the Dirac point approximation when the Dirac points are treated identically, anisotropic effects have been completely ignored at low Fermi energies. Some of the anisotropic effects are raised by increasing the Fermi energy up to the range of trigonal warping limit. However, even at low Fermi energies, band anisotropy of the system manifests itself in the dielectric function $\varepsilon (\overrightarrow{q})$, at least at the range of inter-valley transitions where $q\sim |{\overrightarrow{K}}_{D}-{\overrightarrow{K}}_{D}^{\prime} |$ in which ${\overrightarrow{K}}_{D}$ and ${\overrightarrow{K}}_{D}^{\prime}$ are different Dirac points. At the level of low Fermi energies, the band energy of the system is reduced to a cone-like dispersion. In this case, the Fermi level is identified with a symmetric circle around the Dirac points known as Fermi circle. For a given transferred momentum $\overrightarrow{q}$, different Dirac points have not the same contribution in this type of the physical quantities.This could be considered as another type of anisotropic behaviors in wave number dependent quantities that originate from non-identical contribution of the Dirac points. This work attempts to provide some insight into the limitations of equivalent treatment of the Dirac points. Results of the current work emphasize the need for a systematic revision of identical treatment of the Dirac points in different types of quantities. Specifically, we analyze the robustness of the band anisotropy in graphene and other honeycomb systems which manifests itself in the dielectric function and Friedel oscillations of the system. To achieve this goal, random phase approximation (RPA)^24^ is employed beyond the Dirac point approximation. In this case, at low Fermi energies, the validity of identical contribution of the Dirac points in the dielectric function could be examined within this approach.

It is obvious that the contribution of the nonlinear part of the energy dispersion at high Fermi energies could be given beyond the Dirac point approximation. The exact contribution of each Dirac point can be taken into account when the calculations are performed beyond the Dirac point approximation numerically. It can be shown that even at low Fermi energies the band induced anisotropy could be observed in graphene-like materials. Meanwhile the anisotropic effects which have been observed at low Fermi energies have nothing to do with the nonlinear part of the energy dispersion which is available beyond the Dirac point approximation. This could be understood if we consider that band anisotropy is present both at high and low energy limits.

Friedel oscillation has been reported for graphene using low energy effective Hamiltonian which relies on the Dirac-cone approximation^25–28^. It is important to note that the information about the possible topological phase transitions could be captured by Friedel oscillations. Results of the Friedel oscillations in silicene demonstrates that there is a connection between the Friedel oscillations and topological phase transition^29^. In this work, calculations have been performed beyond the Dirac point approximation in which all possible types of the band anisotropy, including the nonlinear and linear parts of the spectrum, could be considered. Meanwhile, the linear dispersion at Dirac points and even existence of Dirac cones in silicene is being seriously debated^30,31^. Having been motivated by the mentioned points, we have performed current numerical study to obtain a better understanding about the limitations of the Dirac point approximation in graphene-like systems.

## Methods

Graphene-like materials are honeycomb lattice structures. Meanwhile, the SOC of buckled honeycomb structures contains parallel and perpendicular terms. The Hamiltonian of the buckled honeycomb lattice in tight-binding approximation in the presence of SOCs can be written as

1 $$\begin{array}{rcl}H & = & -t\sum _{\langle ij\rangle \alpha }{c}_{i\alpha }^{\dagger }{c}_{j\alpha }+i{t}_{SO}\sum _{\langle \langle ij\rangle \rangle \alpha \beta }{u}_{ij}{c}_{i\alpha }^{\dagger }{\sigma }_{\alpha \beta }^{z}{c}_{j\beta }-i{t}_{intR}\sum _{\langle \langle ij\rangle \rangle \alpha \beta }{\mu }_{ij}{\hat{c}}_{i\alpha }^{\dagger }{(\overrightarrow{\sigma }\times {\overrightarrow{d}}_{ij})}_{\alpha \beta }^{z}{\hat{c}}_{j\beta }\\  &  & +\,i{t}_{extR}\sum _{\langle ij\rangle \alpha \beta }{\hat{c}}_{i\alpha }^{\dagger }{(\overrightarrow{\sigma }\times {\overrightarrow{d}}_{ij})}_{\alpha \beta }^{z}{\hat{c}}_{j\beta }+l\sum _{i\alpha }{\zeta }_{i}{E}_{z}^{i}{\hat{c}}_{i\alpha }^{\dagger }{\hat{c}}_{i\alpha }\end{array}$$

where the operator ${c}_{{ja}}^{\dagger }({c}_{j\alpha })$ creates (annihilates) an electron with spin *α* at site *j* and *t* is the nearest neighbor hopping amplitude. The values of these parameters for different materials are given in Table 1. The *t*_*SO*_ is the spin-orbit induced next-nearest neighbor hopping, ${u}_{ij}={\overrightarrow{d}}_{i}\times {\overrightarrow{d}}_{j}/|{\overrightarrow{d}}_{i}\times {\overrightarrow{d}}_{j}|$ where ${\overrightarrow{d}}_{i}$ and ${\overrightarrow{d}}_{j}$ are the two nearest bonds that connect the next-nearest neighbors, Where *u*_*ij*_ = 1 if the next-nearest neighbor hopping is counterclockwise and *u*_*ij*_ = −1 when it is clockwise with respect to the positive *z* axis^32^. The 〈〈*ij*〉〉 run over all the next-nearest neighbor hopping sites and $\overrightarrow{\sigma }$ is the Pauli matrix. *t*_int*R*_ and *t*_*extR*_ are the strength of intrinsic and extrinsic Rashba SOCs respectively and *μ*_*ij*_ = +1(−1) stands for the A (B) site. *l* is the distance between the two sub-lattice planes in the buckled structures. *ζ* = +1(−1) for the A (B) site and *E*_*z*_ is the applied electric field perpendicular to the plane. The strength of the external Rashba coupling can be manipulated by an external gate voltage. The extrinsic Rashba coupling arises as a result of the inversion symmetry breaking due to an applied perpendicular electric field or interaction with substrate^33^.

**Table 1:** Lattice constant and Energy scales for graphene and other buckled honeycomb materials^8,55–57^.

material	*a*	*t*	*t*_*SO*_	*t*_*int*_ *R*	*l*
silicene	3.86 Å	1.6 eV	0.75 meV	0.46 meV	0.23 Å
germanene	4.02 Å	1.3 eV	8.27 meV	7.13 meV	0.33 Å
graphene	2.46 Å	2.8 eV	0.00114 meV	—	0

**Figure 1:**
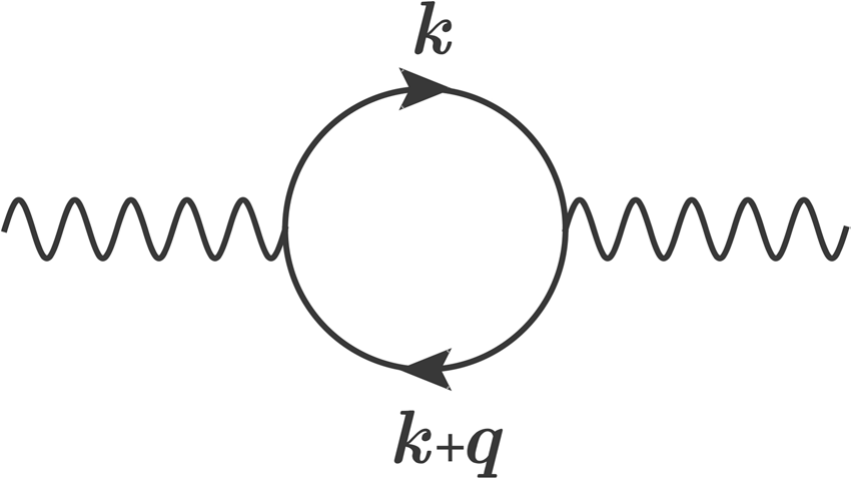
The bare polarization bubble diagram corresponding to Eq. (3).

Dielectric function, screening of charged impurities and also dynamical polarization which gives collective excitations could be captured by the polarization function Π(*ω*, *q*). Dielectric function and collective density oscillations of an electron liquid (plasmons), have been observed in different metals and superconductors^34,35^. At the static limit (*ħω* = 0) polarization function gives the screening behavior of the coulomb potential. The dielectric function is relevant to plasmonic studies. Meanwhile, the transport and phonon spectra are also another relevant fields^36^. The electron-electron interaction has been considered within the random phase approximation characterizes by the density-density correlation function or polarization function (Fig. 1)^24–26,35,37–42^. In this approach dielectric function is given by

2 $$\varepsilon (\omega ,\overrightarrow{q})=1-V(q){\rm{\Pi }}(\omega ,\overrightarrow{q})$$

where *V*(*q*) is the 2D Coulomb potential, *V*(*q*) = 2*πe*^2^/*q*. Within the Dirac point approximation an effective Coulomb potential could be employed in which *V*(*q*) = 2*πα*/*q* and *α* is the ratio of coulomb to kinetic energy and named effective fine structure constant where it has been considered to be *α* = *e*^2^/(*ħε*_0_*v*_*F*_) where *ε*_0_ is the bare dielectric constant and *v*_*F*_ is the Fermi velocity^22^. Unlike to the graphene where the value of fine structure constant could be determined experimentally in different substrates^43^, for other buckled honeycomb structures one can set *α* = 0.8^22^. The polarization function in one loop approximation is calculated directly from the bubble diagram that shown in Fig. (1).

3 $$\begin{array}{rcl}{\rm{\Pi }}(\omega ,\overrightarrow{q}) & = & \sum _{ss^{\prime} kk^{\prime} }\frac{{f}_{k}^{s}-{f}_{k^{\prime} }^{s^{\prime} }}{\omega +{E}_{k}^{s}-{E}_{k^{\prime} }^{s^{\prime} }}| < k^{\prime} {\lambda }_{k^{\prime} }^{s^{\prime} }|{e}^{iq\mathrm{.}r}|k{\lambda }_{k}^{s} > {|}^{2}\\  & = & \sum _{ss^{\prime} k\in BZ}\frac{{f}_{k}^{s}-{f}_{k+q}^{s^{\prime} }}{\omega +{E}_{k}^{s}-{E}_{k+q}^{s^{\prime} }}{F}_{s^{\prime} s}(\overrightarrow{k}+\overrightarrow{q},\overrightarrow{k}),\end{array}$$

All of the results of the present study have been obtained from the Eq. 3 beyond the Dirac point approximation. Where the summation has been performed over the full Brillouin zone (BZ) and all of the eigenstates in which ${f}_{k}^{s}=\frac{1}{\exp \beta ({E}_{k}^{s}-{E}_{F})+1}$ is the Fermi distribution function, and *E*_*F*_ is the Fermi energy. The form factor is given by ${F}_{s^{\prime} ,s}(\overrightarrow{k}^{\prime} ,\overrightarrow{k})=| < k^{\prime} {\lambda }_{k^{\prime} }^{s^{\prime} }|{e}^{iq\mathrm{.}r}|k{\lambda }_{k}^{s} > {|}^{2}=| < k^{\prime} {\lambda }_{k^{\prime} }^{s^{\prime} }|k{\lambda }_{k}^{s} > {|}^{2}{\delta }_{\overrightarrow{k}^{\prime} ,\overrightarrow{k}+\overrightarrow{q}}$ in which $|k{\lambda }_{k}^{s} > =|k > \otimes |{\lambda }_{k}^{s} >$ are the eigenstates of the Hamiltonian where $|{\lambda }_{k}^{s} >$ is the eigenstate in the spin and pseudo-spin subspaces and *s* = 1.. 4 is the band index. ${\delta }_{\overrightarrow{k}^{\prime} ,\overrightarrow{k}+\overrightarrow{q}}$ represents the momentum conservation for contributing transitions.

On the other hand, within the generally used Dirac point approximation the Eq. 3 is reduced to^26^

4 $${\rm{\Pi }}(\omega ,\overrightarrow{q})=g\sum _{ss^{\prime} k\in FC}\frac{{f}_{k}^{s}-{f}_{k+q}^{s^{\prime} }}{\omega +{E}_{k}^{s}-{E}_{k+q}^{s^{\prime} }}{F}_{s^{\prime} s}(\overrightarrow{k}+\overrightarrow{q},\overrightarrow{k})$$

where *g* is valley degeneracy factor and the summation runs around a single Dirac point. *k* ∈ *FC* indicates that the integration should be performed within the Fermi circle (FC) of a given Dirac point. It should be noted that in this relation the degeneracy factor, *g*, implies identical contribution of the different Dirac points in the polarization function at given $\overrightarrow{q}$. As discussed before, the valley degeneracy could result in equivalent contribution of the Dirac points in scalar quantities such as total energy. Consequently, calculation of wave-vector dependent quantities should be performed beyond the Dirac point approximation even at low Fermi energies.

When the integration is reduced to the Fermi circle of a single Dirac point the contribution of the inter-valley transitions is ignored automatically. Inter-valley transitions could take place when the transferred momentum, *q*, satisfies $q\sim \,|{\overrightarrow{K}}_{D}-{\overrightarrow{K}}_{D}^{\prime} |$. At zero Fermi energy the anisotropy of the dielectric function results from this type of transitions.

Within a second-order perturbation approach it has been realized that the exchange interaction of the localized spins *S*_1_ and *S*_2_ with the conducting electrons results in an effective magnetic interaction between these localized magnetic moments known as RKKY interaction given by^44^ *H*_*RKKY*_(*r*) = *JS*_1_.*S*_2_Π(*r*) in which *J* is the exchange coupling constant between the conducting electrons and localized magnetic moments and Π(*r*) is the Fourier transform of the *k*-space polarization function Π(*q*). Therefore, the characteristic properties of this interaction could be captured by the polarization function of the mediating electrons. Accordingly, it is expected that the anisotropy of the polarization function could manifest itself in the anisotropy of the RKKY interaction as it appears in the dielectric function.

The polarization function could be separated into the inter-band (if *s* ≠ *s*′) and the intra-band (if *s* = *s*′) contributions^41^. In addition each of these contributions could be classified as intra-valley and inter-valley transitions correspond to different ranges of transferred momentum, $\overrightarrow{q}$, i.e. $q\le {k}_{F} < |{\overrightarrow{K}}_{D}-{\overrightarrow{K}}_{D}^{\prime} |$ and $q\sim \,|{\overrightarrow{K}}_{D}-{\overrightarrow{K}}_{D}^{\prime} |$ respectively (where *k*_*F*_ is the radius of the Fermi circle and ${\overrightarrow{K}}_{D}$, ${\overrightarrow{K}}_{D}^{\prime}$ are different Dirac points). The static polarization function is of particular importance as it determines the screened potential of a charge impurity which has been assumed to be substituted at a given *A*-site. The screening particle density *δn*(*r*) due to the central impurity *Ze* is,

5 $$\delta n(\overrightarrow{r})=Ze\frac{1}{{(2\pi )}^{2}}\int [\frac{V(\overrightarrow{q}){\rm{\Pi }}(\mathrm{0,}\,\overrightarrow{q})}{\varepsilon (\mathrm{0,}\,\overrightarrow{q})}]\exp (i\overrightarrow{q}\mathrm{.}\overrightarrow{r}){d}^{2}q\mathrm{.}$$

It is really important to note that the above integration goes beyond the limit of intra-valley transitions (the radius of Fermi circle) and therefore the contribution of the inter-valley transitions should be included. This means that the pattern of Friedel oscillations requires the whole information of the static response function in the first Brillouin zone. Accordingly, inter-valley transitions between the different Dirac cones should be included. This type of transitions cannot be captured within the single Dirac cone approximation.

## Non-identical contribution of different Dirac cones

**Figure 2:**
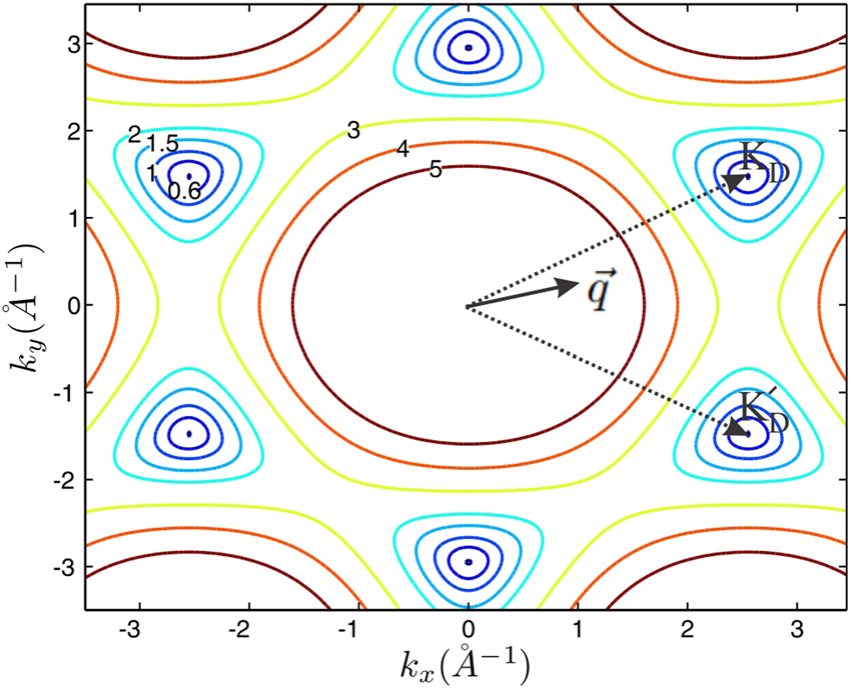
Dirac points of monolayer graphene and Fermi curves at different Fermi energies. Fermi contours have been depicted for *E*_*F*_ = 0.6 *eV*, 1.0 *eV* 1.5 *eV*, 2.0 *eV*, 3.0 *eV*, 4.0 *eV* and 5.0 *eV*. For a given wave vector $\overrightarrow{q}$ the contribution of the different Dirac points on $\varepsilon (\overrightarrow{q})$ strictly depends on the orientation and position of the $\overrightarrow{q}$ with respect to the six Dirac vectors. Trigonal warping of the Fermi curves at different Fermi energies has also indicated in this figure. Single Dirac cone approximation could take into account the anisotropic effects comes from the trigonal warping of a single Fermi curve, however, since the orientation of the deformed Fermi curves are not the same, the anisotropic contribution of the other cones are not identical.

As depicted in Fig. (2) at low Fermi energies the Fermi curves have been appeared as separated islands around each Dirac point. Therefore, the amount of the dielectric and polarization functions in a given $\overrightarrow{q}$ wave number have significantly been determined by the orientation of the wave number with respect to the Dirac points position vectors. This anisotropy of the *q*-space is reflected in the real space quantities such as Friedel oscillations.

One might conclude that the circular shape of the Fermi contours around the Dirac points implies that all of the physical observables of the system should be isotropic as long as the Dirac point approximation is valid. However it should be considered that the isotropic form and circular shape of the Fermi contours around each of the Dirac points cannot result in isotropic properties at least when the inter-valley transitions are taken into account. Accordingly it is important to note that the results of present study cannot be compared with the results that have been obtained within the single-valley Dirac point approximation^45^.

When ${E}_{F} > 0$ both of the intra-valley and inter-valley transitions result in anisotropic dependence of the dielectric function in *q*-space; therefore, different directions of a given transferred momentum, *q*, have not identical contribution even when the Dirac points are degenerate. Accordingly, the conventional single-valley Dirac point approximation could not describe all of the physics of the vector dependent parameters at low wave-length limit. This could also result in anisotropic electric and thermal conductivity in graphene-like materials in the presence of short range scatterers in which all of the intra-valley and inter-valley scatterings are possible.

At non-zero Fermi energies the intra-valley transitions could take place within the range of *q* ≤ *k*_*F*_. Meanwhile we have assumed that the Fermi energy is still low enough where the linear dispersion relation of the Dirac cone is valid at the vicinity of the Fermi energy. It can be shown that both of the intra-valley (*q* ≤ *k*_*F*_ for this case) and inter-valley transitions should be considered as anisotropic contributions in the dielectric function. In this case, since the inter-band transitions ($|k{\lambda }_{k}^{s} > \to |k^{\prime} {\lambda }_{k^{\prime} }^{s^{\prime} } > s\ne s^{\prime}$) are absent in the static limit (*ħω* = 0) all of the contributing terms (both intra-valley and inter-valley transitions) are intra-band. Consequently, the contribution of $q\sim 0$ transitions in the static dielectric function decreases by increasing the Fermi energy. It can be shown that for *E*_*F*_ ≠ 0 we have ${F}_{ss^{\prime} }(\overrightarrow{k}+\overrightarrow{q},\overrightarrow{k})=0$ when *q* = 0 and *s* ≠ *s*′.

**Figure 3:**
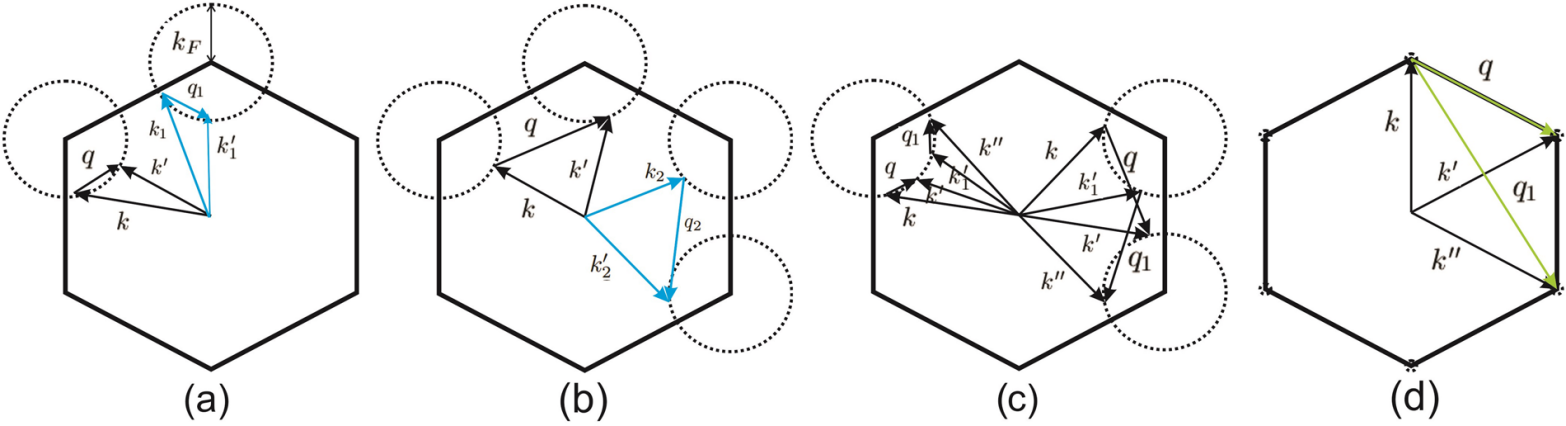
Intra-valley (**a**) and inter-valley (**b**) transitions for a given transferred momentum $\overrightarrow{q}$. Dashed circles indicate the Fermi circles of the honeycomb system in the absence of the spin-orbit couplings. When the momentum conservation rule is satisfied for $\overrightarrow{q}$ the initial and final states should be placed on the Fermi circles. In this case, the contribution of the given states (black vectors) is identical with the contribution of the sixfold-rotated states (cyan vectors). One can imagine about another type of possible transitions (**c**) with constant value of the transferred momentum *q* = *q*_1_ between the equi-energy states ${E}_{k}^{s}={E}_{k^{\prime} }^{s}={E}_{{k^{\prime} }_{1}}^{s}={E}_{k^{\prime \prime} }^{s}$ where the corresponding pair vectors ($\overrightarrow{q}$ ${\overrightarrow{q}}_{1}$), ($\overrightarrow{k}$ $\overrightarrow{k}{^{\prime} }_{1}$) and ($\overrightarrow{k^{\prime} }$ $\vec{k^{\prime \prime} }$) are not related by sixfold symmetry operators e.g. ${ {\mathcal R} }_{2\pi \mathrm{/6}}^{n}\overrightarrow{q}\ne {\overrightarrow{q}}_{1}$. It can be shown that form factor of these transitions are different i.e. *F*(*k*, *k*′) ≠ *F*(*k*′_1_, *k*′′). At zero Fermi energy (**d**) i.e. when *k*_*F*_ = 0 intra-valley transitions occur at *q* = 0 which result in central peak of the dielectric function (has been shown in the next section). However inter-valley transitions (light green vectors) are still the source of anisotropy of the dielectric function.

In the case of intra-valley transitions initial and final states $\overrightarrow{k}$ and $\overrightarrow{k^{\prime} }$ belong to the same Dirac valley cone while in the inter-valley transitions $\overrightarrow{k}$ and $\overrightarrow{k^{\prime} }$ belong to different Dirac cones (Fig. 3(a) and (b)). The momentum conservation rule for each transition between the states $\overrightarrow{k}$ and $\overrightarrow{k^{\prime} }$ with transferred momentum $\overrightarrow{q}$ could be satisfied when $\overrightarrow{k}$ and $\overrightarrow{k^{\prime} }$ sweep the Fermi circles as shown in Fig. 3. Where intra-valley transitions are characterized by 0 ≤ *q* ≤ *k*_*F*_ while inter-valley transitions are identified by $q\sim |{\overrightarrow{K}}_{D}-{\overrightarrow{K}}_{D}^{\prime} |$ where *k*_*F*_ is the radius of the Fermi circle. ${\overrightarrow{K}}_{D}$ and ${\overrightarrow{K}}_{D}^{\prime}$ are different Dirac points as shown in Fig. 3(a) and (b).

In the absence of the SOCs, it can be shown that due to this six-fold band rotational symmetry of the system if the transition rule is satisfied for a given transferred momentum ($\overrightarrow{q}$) it will also be satisfied for the sixfold rotated wave number ${ {\mathcal R} }_{2\pi \mathrm{/6}}^{n}\overrightarrow{q}$ (Fig. 3(a) and (b)). In which ${ {\mathcal R} }_{2\pi \mathrm{/6}}$ is the sixfold rotation operator one can write ${\delta }_{\overrightarrow{k}+\overrightarrow{q},\overrightarrow{k^{\prime} }}={\delta }_{{ {\mathcal R} }_{2\pi \mathrm{/6}}^{n}\overrightarrow{k}+{ {\mathcal R} }_{2\pi \mathrm{/6}}^{n}\overrightarrow{q}\mathrm{,\ }{ {\mathcal R} }_{2\pi \mathrm{/6}}^{n}\overrightarrow{k^{\prime} }}={\delta }_{{\overrightarrow{k}}_{n}+{\overrightarrow{q}}_{n},{\overrightarrow{k^{\prime} }}_{n}}$. Meanwhile, the form factor of is also invariant under the sixfold rotations ${F}_{s^{\prime} s}(\overrightarrow{k}+\overrightarrow{q},\overrightarrow{k})={F}_{s^{\prime} s}({ {\mathcal R} }_{2\pi \mathrm{/6}}^{n}\overrightarrow{k}+{ {\mathcal R} }_{2\pi \mathrm{/6}}^{n}\overrightarrow{q},{ {\mathcal R} }_{2\pi \mathrm{/6}}^{n}\overrightarrow{k})$. In both cases, i.e. for inter-valley and intra-valley transitions band symmetry of the honeycomb structures manifests itself as ${E}_{\overrightarrow{k}}^{s}={E}_{{ {\mathcal R} }_{2\pi \mathrm{/6}}^{n}\overrightarrow{k}}^{s}$. Therefore Eq. 3 reveals that for a given transferred momentum, $\overrightarrow{q}$, satisfying the transition rule $\overrightarrow{k}=\overrightarrow{k}^{\prime} -\overrightarrow{q}$. The contribution of the $\overrightarrow{k}\to \overrightarrow{k}^{\prime}$ scattering in the dielectric function is identical with the contributions of the ${ {\mathcal R} }_{2\pi \mathrm{/6}}^{n}\overrightarrow{k}\to { {\mathcal R} }_{2\pi \mathrm{/6}}^{n}\overrightarrow{k}^{\prime}$ scatterings for both of the inter-valley and intra-valley transitions. In other words, it could be inferred that $\varepsilon (\overrightarrow{q})=\varepsilon ({ {\mathcal R} }_{2\pi \mathrm{/6}}\overrightarrow{q})$ $=\,\varepsilon ({ {\mathcal R} }_{2\pi \mathrm{/6}}^{2}\overrightarrow{q})$ $=\ldots =\,\varepsilon ({ {\mathcal R} }_{2\pi \mathrm{/6}}^{5}\overrightarrow{q})$.

The first consequence of the above argument is that the different Fermi curves of each Dirac point have not identical contribution on the dielectric function at a given $\overrightarrow{q}$ wave number. When $\overrightarrow{q}$ satisfies the transition rule for a specific Dirac cone (for example in an intra-valley process) this rule will be satisfied for ${ {\mathcal R} }_{2\pi \mathrm{/6}}^{n}\overrightarrow{q}\,(n=1\,\mathrm{...}\,\mathrm{5)}$ at other Dirac cones where $\overrightarrow{q}$ itself could not satisfy the momentum conservation rule or could not give the same contribution at these Dirac cones. Accordingly, the dielectric function should be anisotropic in the *q*−space with sixfold symmetry which was originated from the symmetry of the band structure.

All of the other possible transitions with a given fixed value of the transferred momentum could take place between the isoenergy states as shown in Fig. 3(c). In this group of the transitions, transferred momentum is the same *q* = *q*_1_ (disregarding its direction) and both of the initial final states are located at Fermi circle ${E}_{k}^{s}={E}_{k^{\prime} }^{s}={E}_{{k^{\prime} }_{1}}^{s}={E}_{k^{\prime \prime} }^{s}={E}_{F}$. However, corresponding pair vectors are not related by sixfold symmetry operators i.e. ${ {\mathcal R} }_{2\pi \mathrm{/6}}^{n}\overrightarrow{q}\ne {\overrightarrow{q}}_{1}$, ${ {\mathcal R} }_{2\pi \mathrm{/6}}^{n}\overrightarrow{k}\ne \overrightarrow{k}{^{\prime} }_{1}$ and ${ {\mathcal R} }_{2\pi \mathrm{/6}}^{n}\overrightarrow{k^{\prime} }\ne \overrightarrow{k}^{\prime\prime}$. The transition rule has been satisfied for these transition where we have $\overrightarrow{k}+\overrightarrow{q}=\overrightarrow{k}^{\prime}$ and $\overrightarrow{k}{^{\prime} }_{1}+{\overrightarrow{q}}_{1}=\overrightarrow{k}^{\prime\prime}$. Meanwhile, the form factor of the transitions are not the same *F*(*k*, *k*′) ≠ *F*(*k*′_1_, *k*′′) which implies that the corresponding contributions are not identical (Fig. 3(c)).

When the Fermi energy located at Dirac points i.e. *E*_*F*_ = 0 then, the intra-valley transitions occur just in $\overrightarrow{q}=0$ between different bands (Fig. 3(d)). Unlike the case *E*_*F*_ ≠ 0 the intra-valley contributions are identical at zero Fermi energy. These contributions result in the central peak of the dielectric function. Since the *V*(*q*) = 2*πe*^2^/*q* diverges at *q* = 0. However the inter-valley transitions that occur away from the Γ-point (*q* = 0) result in anisotropic contributions as the previous case.

## Results and Discussions

Due to the foregoing discussions for wave number-dependent or non-scalar quantities such as dielectric function, electric and thermal conductivities, we have to concern about the position of the Dirac points relative to the direction of the characteristic vector of the physical quantity (such as transfered momentum) even when the Dirac point approximation is valid. For sharp scattering potentials, we have to consider the inter-valley transitions in the calculation of these quantities. Within the Dirac point approximation, the integration over the state-resolved contributions is generally performed over a single Fermi circle. This could be a correct approach when we assume the identical contribution of each Dirac point and ignore the inter-valley transitions. In this case, if we put aside the Dirac point approximation and perform the integration over the whole Brillouin zone the correct contribution of each Dirac point could be obtained.

The main limitations for the use of the Dirac point approximation has been discussed within the current work. Non-identical contribution of the Dirac points results in anisotropic dielectric function in *k*-space. Moreover, the anisotropy of the dielectric function leads to anisotropic Friedel oscillation in graphene-like materials. Increasing the Rashba coupling strength cannot results in significant change in the dielectric function and Friedel oscillation. At the level of the Dirac point approximation anisotropy of the band energy and its relevant effects have been ignored. Meanwhile, the anisotropy of single Fermi circle reshaping, which arises by increasing the Fermi energy, could be obtained within the single Dirac cone approximation.

According to the numerical results, two-dimensional graphene like materials show anisotropic Friedel oscillations beyond the Dirac cone approximation even when the Dirac points located at the Fermi level. At first look, it seems that the degeneracy of the K-points results in identical contribution of each Dirac point in all of the physical quantities. However, this is not the case for some of the quantities which directly depend on the direction of the transfered wave number. In this case each of the Dirac points has not identical contribution for this type of the quantities, even when the Dirac point approximation is valid. Meanwhile, since the inter-valley transitions could take place between the different Dirac cones, these type of transitions cannot be captured within the single Dirac cone approximation.

Anisotropic Friedel oscillations in two-Dimensional structures have been observed before^46^. However, in the present case, the anisotropic effects are direct manifestation of non-identical contribution of Fermi circles of different Dirac points in wave number dependent quantities. Some of the physical quantities, such as dielectric function, are given by integration over the Brillouin zone as expressed in Eq. 3. This integration goes beyond the states in which the Dirac point approximation and the linear dispersion relation no longer valid. However, distribution function at low temperatures and Fermi energies picks up the contribution of those states which have been located near to the Dirac points. Nevertheless, isoenergy inter-valley transitions between these states can induce the anisotropic effects in multi-valley structures.

The linear dispersion relation (and therefore circle like Fermi curves) around the Dirac points valid even up to ${E}_{F}\sim 1$ eV in graphene and the thermal transitions at room temperature with *K*_*B*_*T* = 0.025 eV could not induce any considerable contribution from those states which have been located far from the Dirac points. So it seems that the Dirac point approximation could still describe the physics of the honeycomb lattice and the linear dispersion relation around the Dirac points could be employed for the calculation of the dielectric function. This means that the non-linear part of the band structure and the anisotropy that might be induced by this part could be ignored. This is due to the fact that this part of the band structure (which could be considered the energy states with ${E}_{k}^{s} > 1eV$) could not contribute in the isoenergy transitions of the static limit and low Fermi energies. Therefore, the anisotropic effects arise primarily from the inter-valley transitions which cannot be described within the Dirac point approximation.

**Figure 4:**
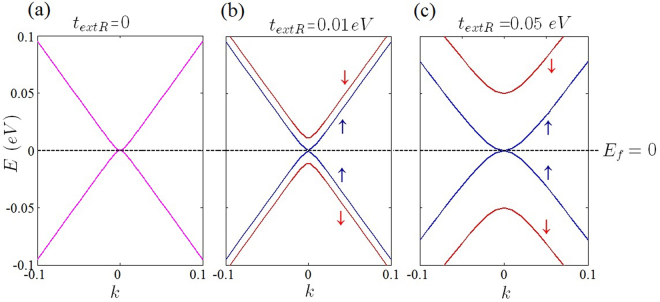
Band structure of gapless graphene at different Rashba couplings.

**Figure 5:**
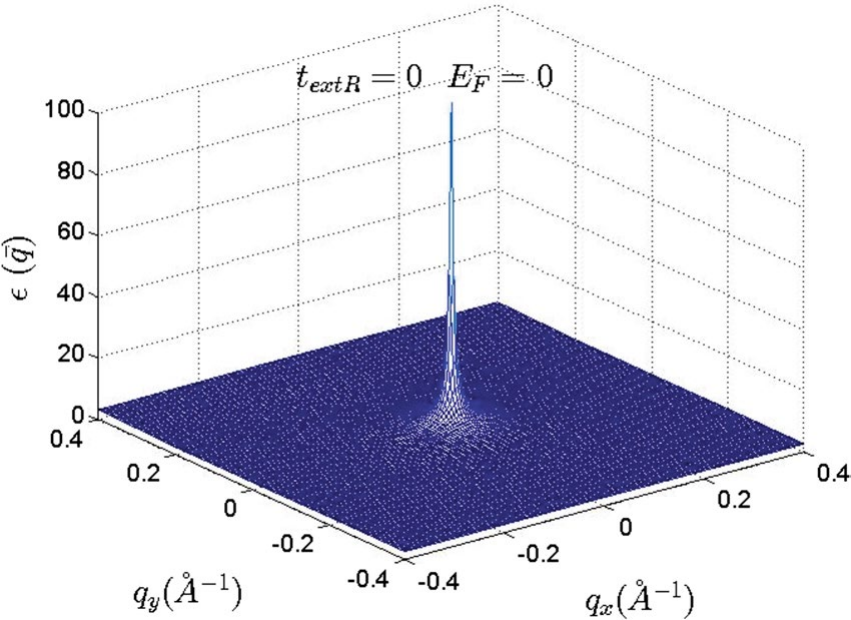
*k*-space dielectric function of monolayer graphene at *t*_*extR*_ = 0. Additionally it was assumed that the intrinsic spin-orbit coupling has been also negligible. This enables us to compute the net band induced anisotropic effects.

Increasing the Fermi energy results in deformation of circle-like Fermi curves (Fermi-circles) of low Fermi energies around the Dirac points^47^. In this case, the isotropic form of the Fermi circles change into the trigonal-shaped contours (known as trigonal warping effect) and the isotropic form of the Fermi curve around of the Dirac points has totally been removed at high Fermi energies. This type of deformation could results in a new source of anisotropy at high Fermi energies which could be captured within the single cone approximation. Meanwhile, in the current work, which was limited to the low Fermi energies, trigonal warping induced anisotropy has not been considered. At low Fermi energies for gap-less honeycomb structures such as graphene (Fig. (4)) optical transitions around each Dirac points, taking place within a single Fermi circle, have the main contribution in the dielectric function of the honeycomb systems. This manifests itself as a central peak of the dielectric function in the middle of the Brillouin zone (Fig. 5). Beyond the Dirac point approximation the central peak contains the contribution of all of the Dirac points via the inter-band transitions. When the Fermi energy is close to Dirac points (*E*_*F*_ = 0) and at the long wave length limit ($q\ll |{K}_{D}-{K^{\prime} }_{D}$) the occupation factor ${f}_{k}^{s}-{f}_{k^{\prime} }^{s^{\prime} }$ is significant only when the *k* and *k*′ states are close to the Dirac point of different bands (*s* ≠ *s*′). Meanwhile, the Kronecker delta, ${\delta }_{\overrightarrow{k^{\prime} },\overrightarrow{k}+\overrightarrow{q}}$, in the expression of the polarization indicates that the contribution of the Dirac points should be selected by Γ-point $(\overrightarrow{q}=\mathrm{0)}$. Since at this limit the main contribution is due to the intra-valley transitions which take place near the Dirac points in which *k* ≈ *K*_*D*_ and *k*′ ≈ *K*_*D*_, the mentioned Kronecker delta which reflects the momentum conservation, imposes that the contribution of the Dirac points should be manifest themselves at the Γ-point $(\overrightarrow{q}\approx \mathrm{0)}$ of the *q*-space (Fig. 5). The mentioned argument reveals the fact that the central peak of the dielectric function in graphene is exactly sum of all of the intra-valley contributions from each Dirac point. Although the contribution of the intra-valley transitions are dominant at $q\sim 0$, this is not the case for inter-valley transitions within the range of |*K*_*D*_ − *K*′_*D*_| − 2*k*_*F*_ ≤ *q* < |*K*_*D*_ − *K*′_*D*_| + 2*k*_*F*_.

In order to obtain the anisotropic effects which have been induced merely by band energy we switch off both types of the spin orbit couplings. At zero Rashba interaction, both intrinsic and extrinsic spin-orbit couplings are absent. This enables us to obtain the anisotropic effects which could be induced merely by band energy. Dielectric function at zero Rashba coupling and zero Fermi energy (*E*_*F*_ = 0) has been obtained as depicted in Fig. 5.

**Figure 6:**
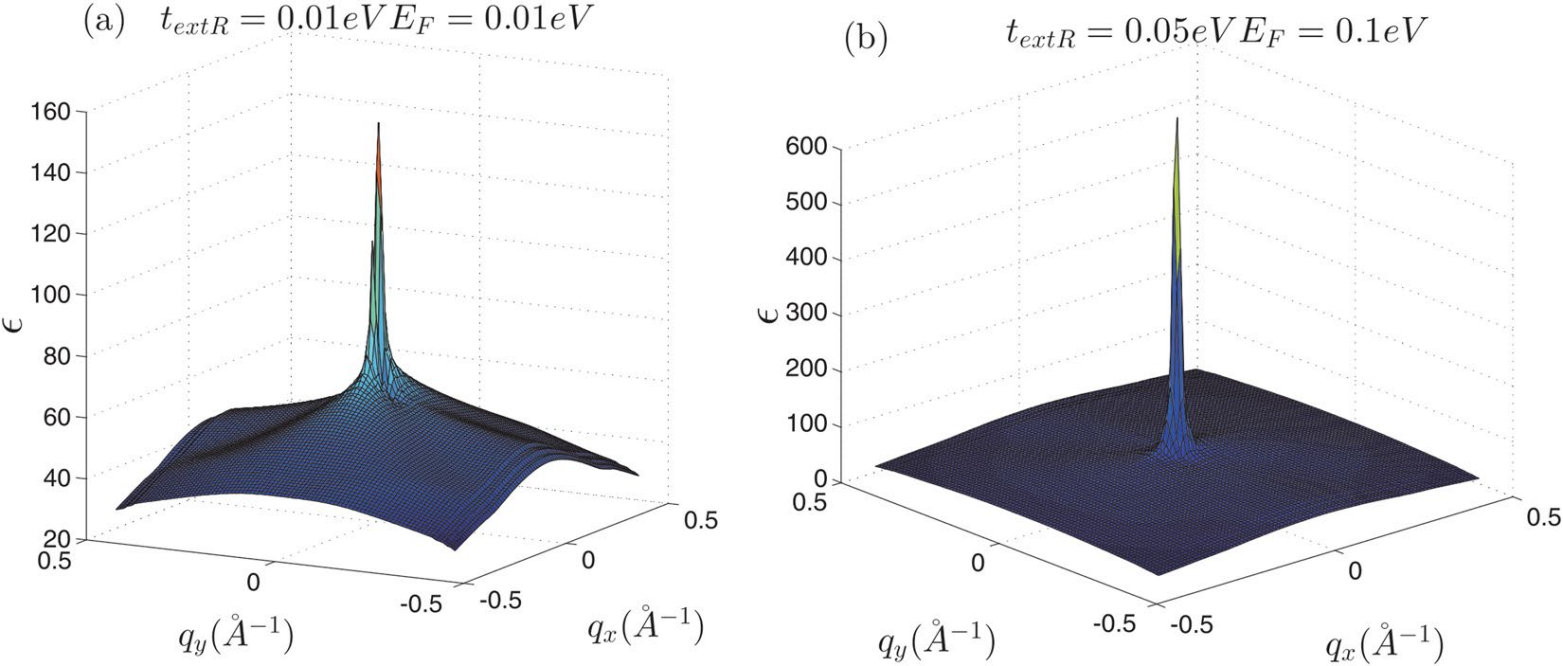
*k*-space dielectric function of monolayer silicene and germanene.

**Figure 7:**
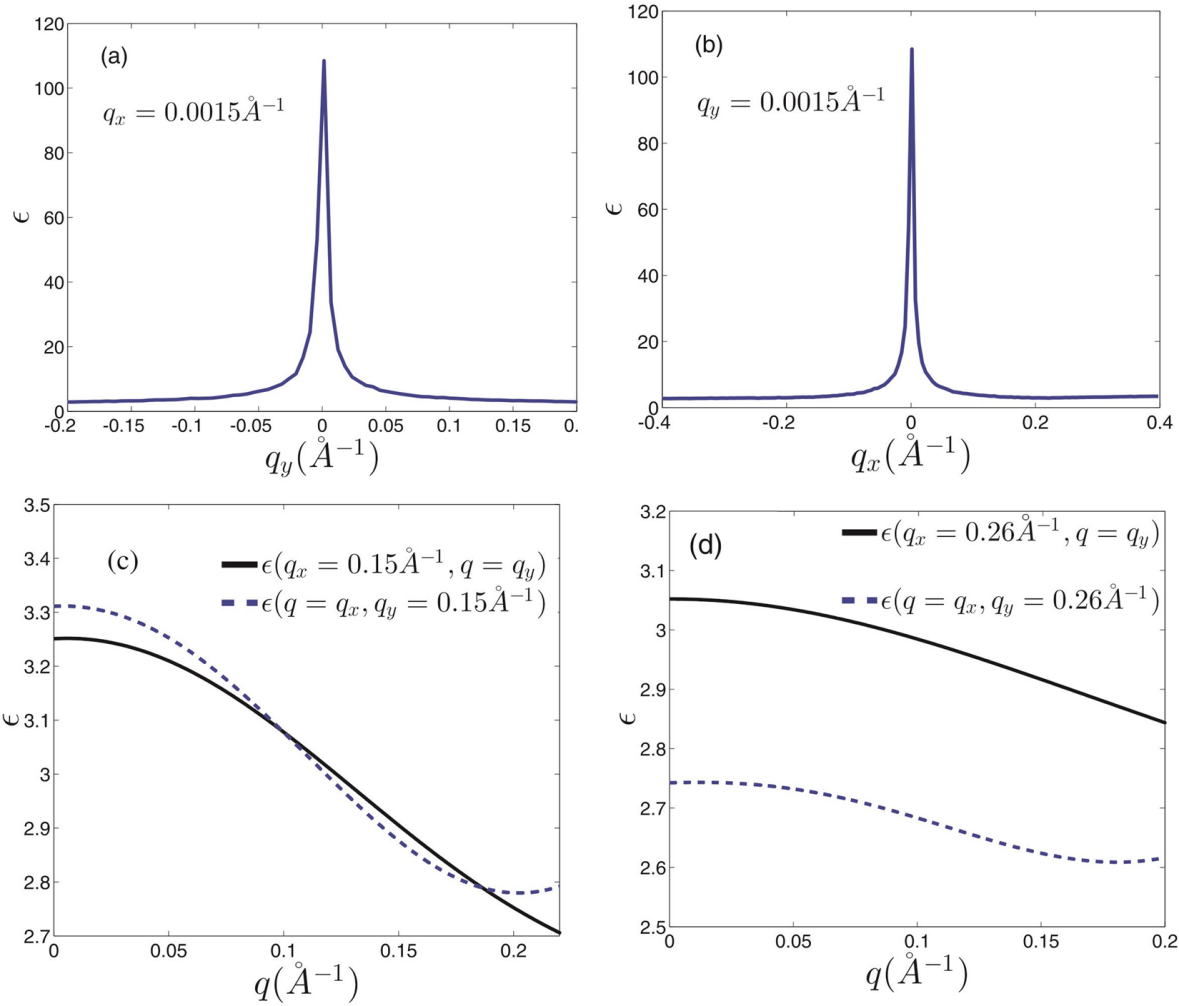
Dielectric function of monolayer graphene at different symmetrically chosen slices for *t*_*extR*_ = 0 in *k*-space. (**a**) At *q*_*x*_ = 0.0015 *Å*^−1^ plane. (**b**) *q*_*y*_ = 0.0015 *Å*^−1^ plane. (**c**) At *q*_*x*_ = 0.15 *Å*^−1^ and *q*_*y*_ = 0.15 *Å*^−1^ slices. (**d**) At *q*_*x*_ = 0.26 *Å*^−1^ and *q*_*y*_ = 0.26 *Å*^−1^ planes. Anisotropic effects appear far away from the origin where the contribution of the inter-valley transitions should be taken into account.

At the first look, it seems that there is no anisotropy in the dielectric function of the honeycomb structures (Figs 5 and 6), however, it should be noted that the anisotropy of the dielectric function has been hidden behind the large central peak at $q\sim 0$. The amount of the dielectric anisotropy is very small in comparison with the value of the dielectric function at the Γ-point. Accordingly, this fact prevents the identification of the directional dependence of the dielectric function. At low wave numbers i.e. in the range of the intra-valley transitions dielectric function seems to be quite isotropic in *q*-space (Fig. 7(a) and (b)). However, far from the central region if we select different symmetric slices of the dielectric surface, the anisotropy of the dielectric function will be evident (Fig. 7(c) and (d)). It can be expected that the anisotropy of the dielectric function would be appeared at $q\ge \,|{\overrightarrow{K}}_{D}-{\overrightarrow{K}}_{D}^{\prime} |$ (Fig. 7(c) and (d)). Dirac point approximation based studies result in completely isotropic dielectric function. At the log wave length limit, the static dielectric function within the Dirac point approximation has been suggested to be *ε*(*q*) = 1 + 2*πe*^2^*D*_0_/*κq*. In which *κ* is the background dielectric constant and *D*_0_ = *D*(*E*_*F*_) is the density of states at Fermi energy^40,48^.

**Figure 8:**
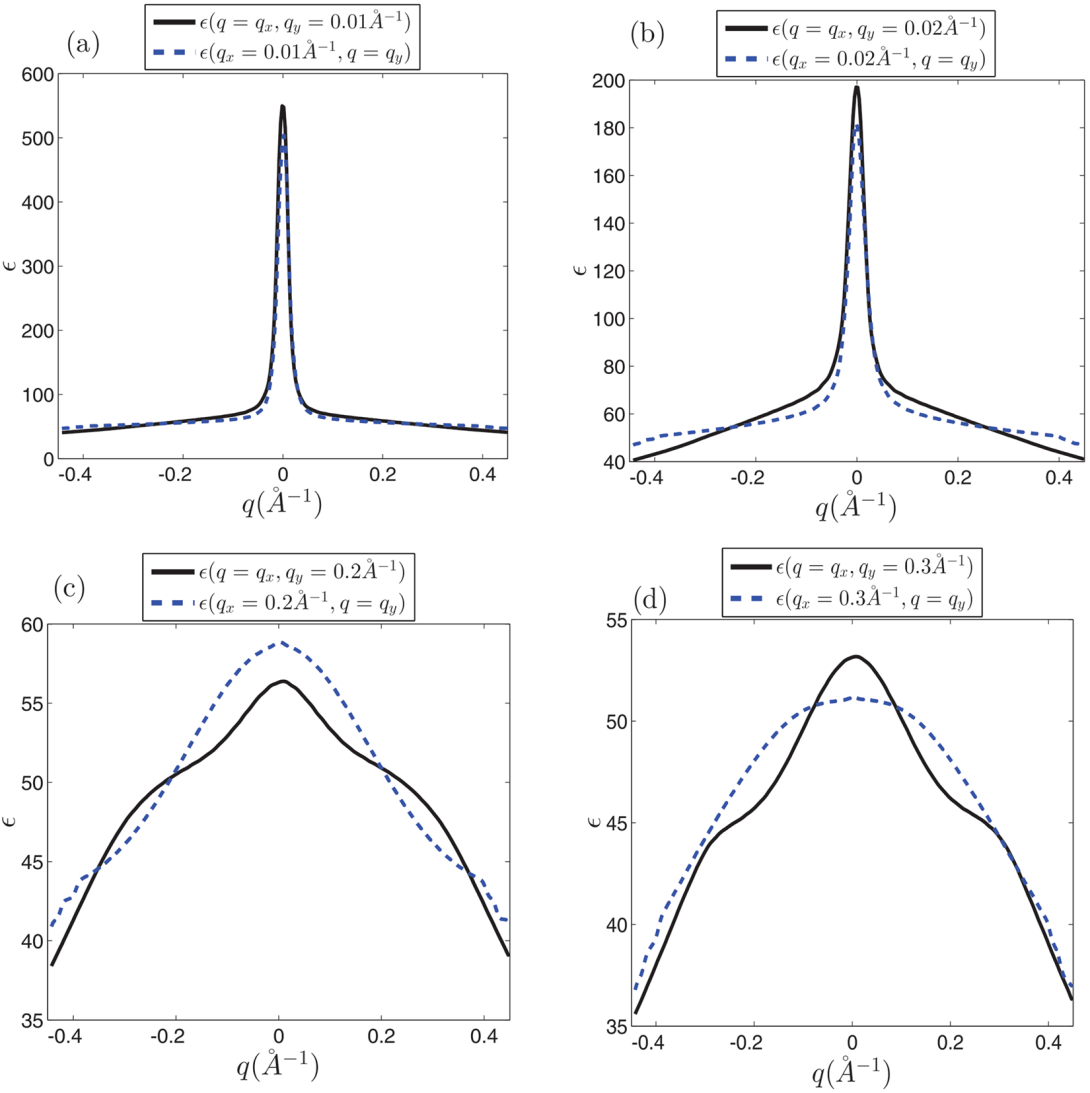
Dielectric function of monolayer silicene at different symmetrically chosen slices for *t*_*extR*_ = 0.01 eV in *k*-space. (**a**) At *q*_*α*_ = 0.01 *Å*^−1^ planes (*α* = *x*, *y*). (**b**) *q*_*α*_ = 0.02 *Å*^−1^ planes. (**c**) At *q*_*α*_ = 0.2 *Å*^−1^ slices. (**d**) At *q*_*α*_ = 0.3 *Å*^−1^ planes. Anisotropic effects appear far away from the origin where the contribution of the inter-valley transitions is dominant.

**Figure 9:**
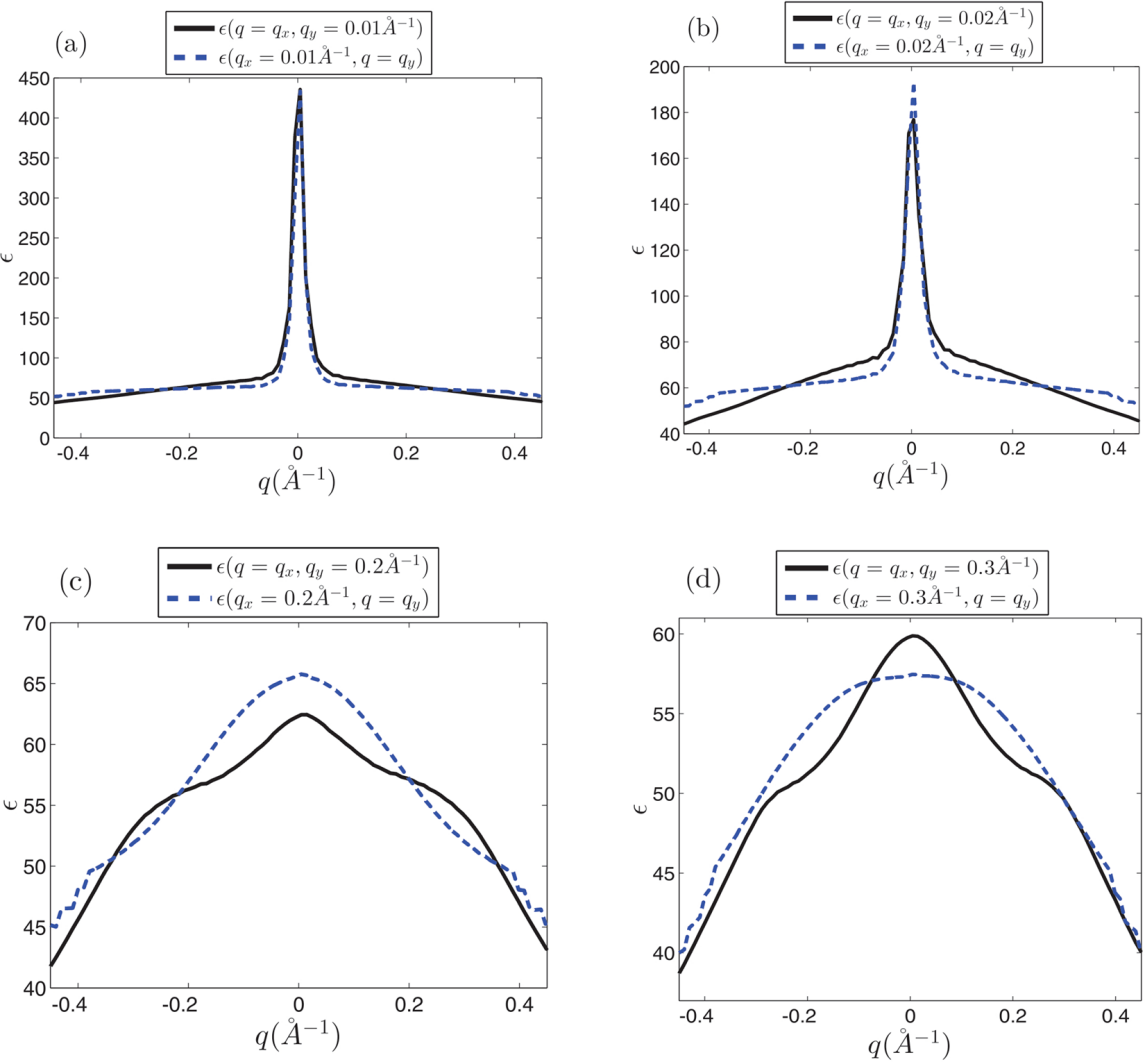
Dielectric function of monolayer germanene at different symmetrically chosen slices for *t*_*extR*_ = 0.05 eV in *k*-space. (**a**) At *q*_*α*_ = 0.01 *Å*^−1^ planes (*α* = *x*, *y*). (**b**) *q*_*α*_ = 0.02 *Å*^−1^ planes. (**c**) At *q*_*α*_ = 0.2 *Å*^−1^ slices. (**d**) At *q*_*α*_ = 0.3 *Å*^−1^ planes. Anisotropic effects appear far away from the origin where the contribution of the inter-valley transitions is dominant.

A similar discussion holds for the case of silicene and germanene where as shown in Figs 8 and 9 dielectric function has completely different behavior on symmetrically chosen slices, especially far enough the Γ point. These figures evidently show that for different directions in the *k*-space behavior of the dielectric function are quite different. This reveals that the contributing inter-valley transitions in finite wave length limit ($q\sim \,|{K}_{D}-{K^{\prime} }_{D}|$) introduce the anisotropic behaviors.

**Figure 10:**
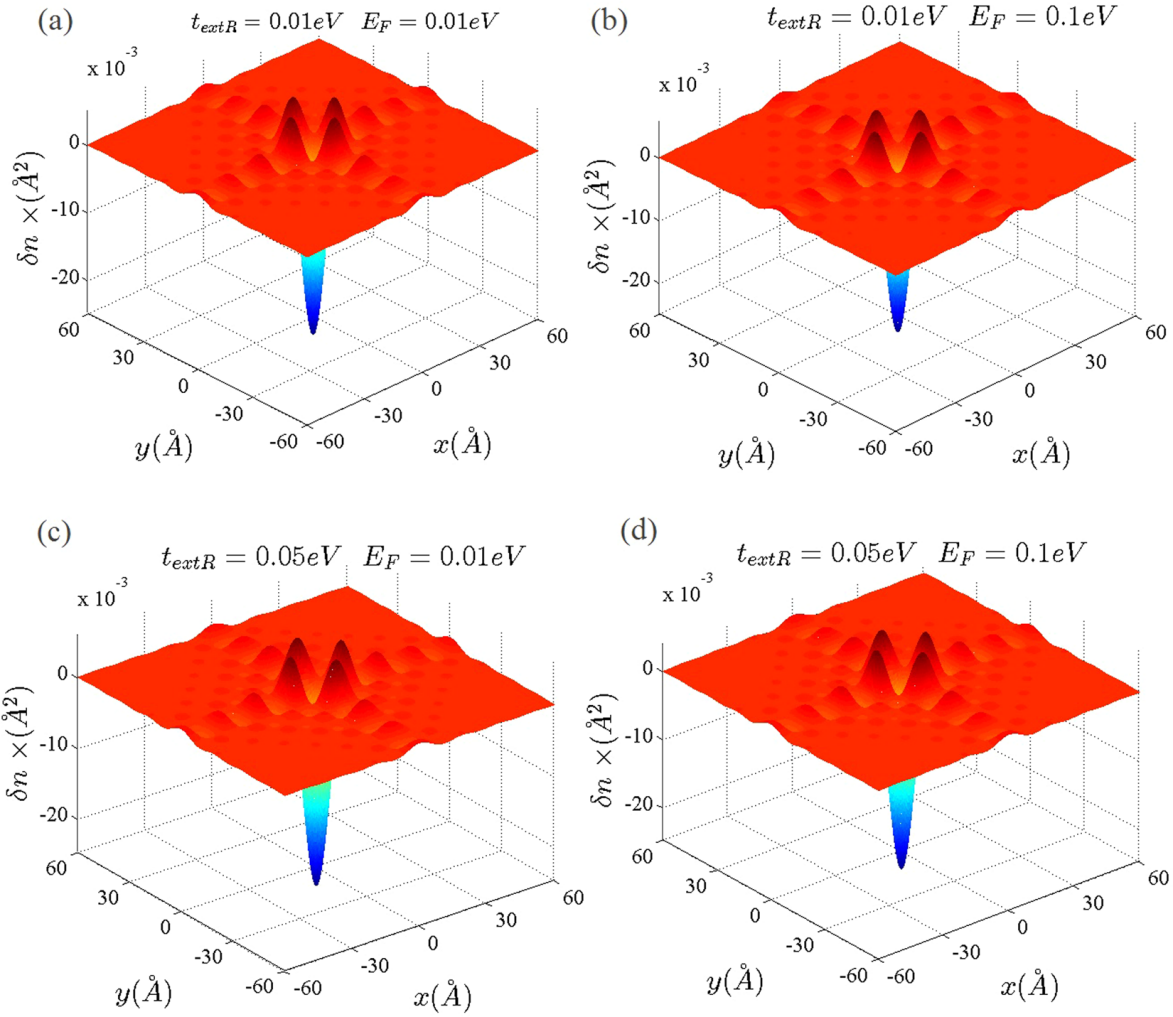
Real space anisotropic Friedel oscillations in monolayer graphene at different Rashba couplings. (**a**–**d**) As shown in these figures the Rashba interaction has not a significant influence on the Friedel oscillations at intermediate Rashba coupling strength.

**Figure 11:**
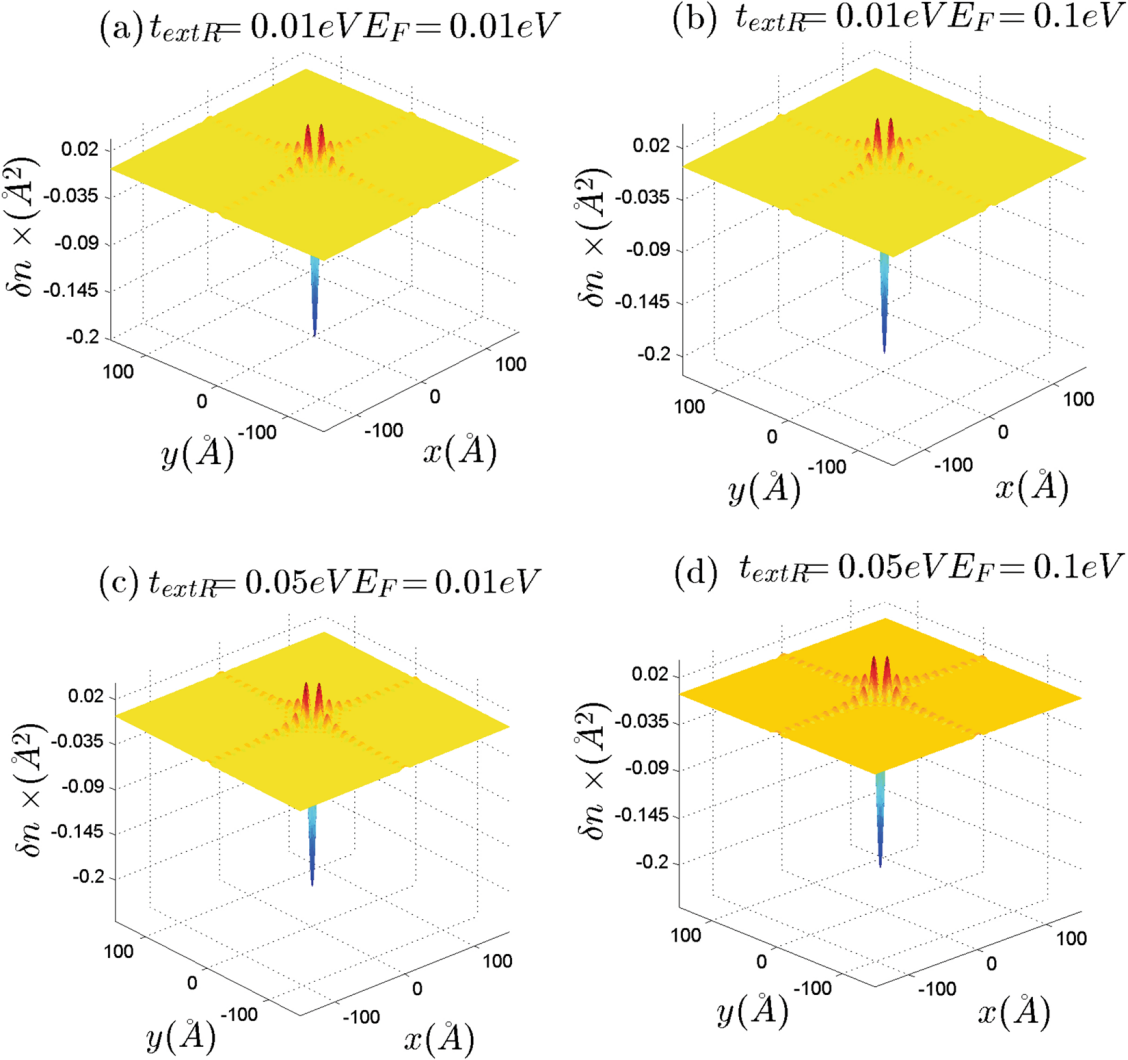
Real space anisotropic Friedel oscillations in monolayer silicene ((**a**) and (**b**)) and germanene ((**c**) and (**d**)) at different Rashba couplings. The Rashba interaction has not a significant influence on the Friedel oscillations.

**Figure 12:**
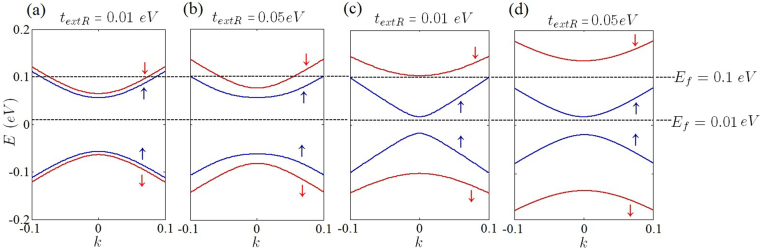
Band structure of silicene (**a**,**b**) and germanene (**c**,**d**) when the system is band insulator in the different values of external Rashba term and Fermi energies. *lE*_*z*_ = 0.06 *eV* where $l{E}_{z} > {t}_{SO}$ for both silicene and germanene.

As discussed before, band induced anisotropic effects (see Fig. 2) have been reflected in the Friedel oscillations of the graphene-like structures illustrated in Figs 10 and 11. In addition, corresponding band structures near the Dirac points and given Fermi levels have been shown in Fig. 12. Increasing the Rashba coupling strength slightly modifies the Friedel oscillations honeycomb structures Figs 10 and 11. This could be explained if we consider relatively large and dominant intrinsic spin-orbit coupling in silicene. As shown in the Fig. (10), the anisotropic Friedel oscillations have been observed even when the Rashba coupling strength is very low or zero. It can be inferred from the results of the current work that the Rashba coupling is less effective in the generation of the anisotropy. Therefore one can conclude that the anisotropy of the dielectric function and Friedel oscillations mainly depends on the anisotropy of the band structure in *k*-space.

There are several studies which have been performed in this field, aiming at an accurate quantitative prediction of dynamical dielectric function, screened charged impurity potential and Friedel oscillations in graphene-like materials. It was realized that the long-distance decay of Friedel oscillations in graphene depends on the symmetry of the scatterer^49^. A faster, $\delta n\sim \mathrm{1/}{r}^{3}$, decay in comparison with conventional 2D electron systems has been observed in Friedel oscillations of a localized impurity inside the monolayer graphene within the Dirac point approximation^26,49^. However, 1/*r* decay has been reported for bilayer graphene^50^ and strong asymmetry and an inverse square-root decay has also been obtained for an anisotropic graphene-like structure when one of the nearest-neighbor hopping amplitudes is different from the others^51^. Recently, in rhombohedral graphene multi-layers, 1/*r* decay has been observed for impurity induced Friedel oscillations^52^. Completely isotropic behavior has been reported for the potential of a screened charged impurity, Friedel oscillations^25–28^ and static dielectric function^40^ within the Dirac point approximation in graphene. Similarly, the Dirac point approximation results in isotropic screened potential of a charged impurity in other graphene-like materials such as silicene and germanene^22^.

The Dirac point approximation based studies give the correct physics of the long wave length limit (*q* ≪ *k*_*F*_) at *E*_*F*_ = 0 where inter-valley transitions could not contribute in the physical processes. In the absence of the spin-orbit couplings by using the massless linear Dirac spectrum it was also shown that short wavelength spatial dependence of the local density of states leads to anisotropic Friedel oscillations which has the form^53^.

6 $$\delta n(r)\sim c(\overrightarrow{r}){\rho }_{0}({E}_{F})\frac{\sin \,\mathrm{(2}{k}_{F}r)}{{r}^{2}}\mathrm{.}$$

In which $c(\overrightarrow{r})$ is the short wavelength spatial dependence factor and *ρ*_0_(*E*) is the density of states. Anisotropic dependence of the Friedel oscillations has been introduced by *c*($\overrightarrow{r}$) factor which was found to be invariant under threefold rotations^53^. However, if the impurity could not produce inter-valley scatterings this factor is reduced to a constant number^53^. Therefore, the anisotropic effects have been removed in the absence of inter-valley transitions^53^. In the current study, we have observed that for finite Fermi energies 0 < *E*_*F*_ ≤ 1 eV intra-valley transitions are the source of the anisotropic behaviors at linear energy dispersion regime.

In the case of the single valley band structures, where all of the transitions are intra-valley transitions, the wave length of the Friedel oscillations is modulated by Fermi wave number. However, it can be easily shown that this is not the case for multi-valley band structures. In which the inter-valley transitions could contribute in the dielectric function. As indicated in Eq. (6) it was expected that the wavelength of the Friedel oscillations should be modulated by the Fermi wave-vector *k*_*F*_^53^. Where the long range behavior of the local density of states has been obtained within the single valley approximation and linear dispersion relation^53^. The possible transfered momentums, *q*, determine the oscillation wavelength of the induced charged and for single valley band structures in two-dimensional systems typical transfered momentum is $q\sim 2{k}_{F}$. However it should be noted that for a typical graphene Fermi energy e.g. *E*_*F*_ = 0.1 eV one can obtain *k*_*F*_ = *E*_*F*_/(*ħv*_*F*_) = 0.0152 *Å*^−1^. Therefore, the oscillation wavelength that corresponds to the intra-valley transitions is about ${\lambda }_{intra}=2\pi /2{k}_{F}\sim 200$ Å. On the other hand, in the present case, the inter-valley transitions with momentum transfer of $q\sim \,|\overrightarrow{K}-\overrightarrow{K}^{\prime} |\pm 2{k}_{F}$ correspond to the oscillations with wavelength of ${\lambda }_{inter}=2\pi /(|\overrightarrow{K}-\overrightarrow{K}^{\prime} |\pm 2{k}_{F})$. The wavelengths of the oscillations in the current work for monolayer graphene are in the following range 7Å $\lesssim {\lambda }_{inter}\lesssim$13 Å that are at the same order of the inter-valley transition wavelengths given by ${\lambda }_{inter}=2\pi /(|\overrightarrow{K}-\overrightarrow{K}^{\prime} |\pm 2{k}_{F})$. For example the distance between two successive Dirac points in graphene is about ${\rm{\Delta }}K=|K-K^{\prime} |\,\sim 1.7$ Å^−1^. Therefore the average momentum transfer between these Dirac points is $q\sim \,|\vec{K}-\vec{K}^{\prime} |/2=0.85$ Å^−1^ then *λ*_*inter*_ = 2*π*/Δ*K* = 7.37 *Å*. Results indicate that the wavelength of the oscillations is less-sensitive to the value of the Fermi energy and Rashba coupling strength. This can be realized if we consider that $|\overrightarrow{K}-\overrightarrow{K}^{\prime} |\,\gg 2{k}_{F}$ for intermediate Fermi energies. Accordingly, the difference of the Friedel oscillations in graphene-like materials characterizes just by the Dirac point wave-vector (${\overrightarrow{K}}_{D}$) of each structure.

Decay rate of the Friedel oscillations are determined by fitting to the numerical results. We have examined several decay rates such as 1/*r*, 1/*r*^2^ and 1/*r*^3^. Numerical fitting shows that the 1/*r* decay rate is much more close to the computational data profile. More precisely decay rate is actually 1/*r*^1+*η*^ where 0 < *η* < 0.2.

**Figure 13:**
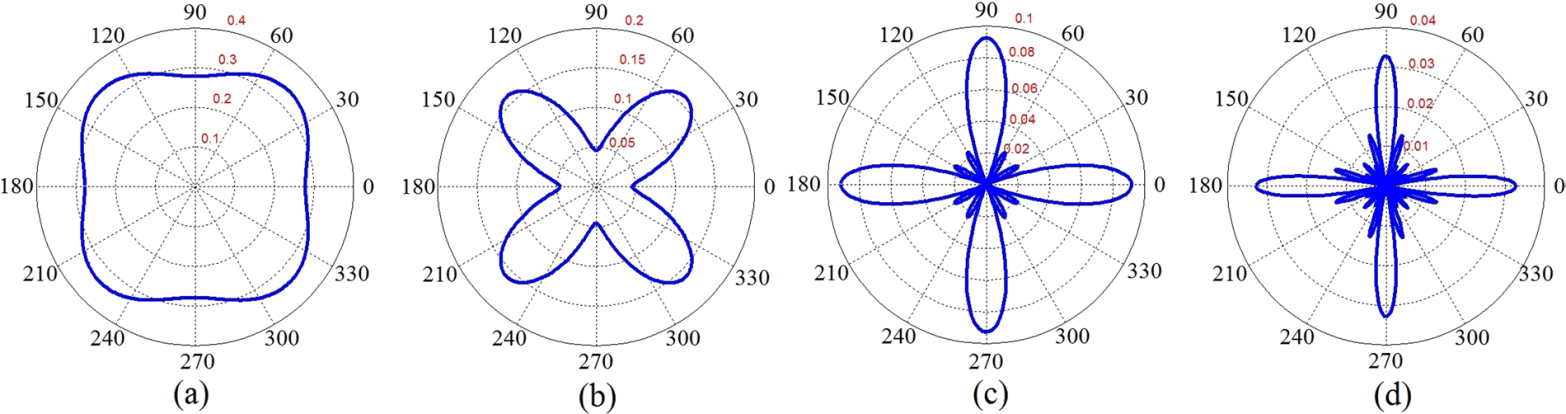
Angular dependence of the normalized graphene Friedel oscillations, *δn*(*r*, *θ*)/*n*_0_, for *t*_*extR*_ = 0, *E*_*F*_ = 0.1*t* and *K*_*B*_*T* = 0 at (**a**) *r* = 4 *Å*, (**b**) *r* = 5 *Å*, (**c**) *r* = 14 *Å* and (**d**) *r* = 25 *Å* in which we have defined ${n}_{0}=\delta n(\overrightarrow{r}=\mathrm{0)}$.

Another important issue about the Friedel oscillations is that how sharp the mentioned density anisotropy really is? In this way, we have obtained the angular dependence of the induced density at different distances as depicted in Fig. (13). As indicated in this figure the anisotropy of the Friedel oscillations increases by distance. It can be realized that the angular dependence of the induced density is so sharp at intermediate distances. This provides more detectable condition for observation of the anisotropy.

Interestingly, it was shown that the Friedel oscillations in graphene have a strong sublattice asymmetry^54^. These calculations have been performed beyond the Dirac point approximation within the Born approximation which can be employed for weak scattering potentials and the stationary phase approximation (SPA) has also been applied for Brillouin zone integrations^54^. Anisotropic Friedel oscillations could also be inferred from the numerical results of the recent work in the absence of the spin-orbit interactions especially over short distances.

Finally, it is important to note that the anisotropy of the dielectric function suggests that the orientation of the bases vectors of the honeycomb lattice could be determined by full optical measurements. Since dynamical dielectric function of the graphene-like materials possibly have the same anisotropic nature, the absorption spectra of honeycomb structures (the imaginary part of the dielectric function) should be anisotropic. Accordingly, the real space orientation of the basis vectors could be explored since the absorption spectra leads to identification of the band energy configuration in k-space.

## Electronic supplementary material

Supplementary File
