# Supplementary material for: Anisotropic Friedel oscillations in graphene-like materials: The Dirac point approximation in wave-number dependent quantities revisited
Source: Sci Rep. 2018 Feb 8;8:2667. doi: 10.1038/s41598-018-19730-2 (PMC5805790; doi:10.1038/s41598-018-19730-2)
Supplement: Supplementary file 1 — Supplementary File [file 41598_2018_19730_MOESM1_ESM.pdf]

Anisotropic Friedel oscillations in graphene-like materials: The Dirac point approximation in wave-number dependent quantities revisited

Tohid Farajollahpour, Shirin Khamouei, Shabnam Safari Shateri, and Arash Phirouznia

SUPPLEMENTARY INFORMATION

TIGHT-BINDING HAMILTONIAN OF GRAPHENE LIKE STRUCTURES

The crystal structure of graphene-like materials is a honeycomb lattice, similar to graphene meanwhile the SOC for buckled honeycomb structures have two parallel and perpendicular terms.

The Hamiltonian of the buckled honeycomb lattice within the tight-binding approximation in the presence of SOCs can be written as

$$H = H_0 + H_{SO} + H_{intR} + H_{extR} + H_{sub}. \tag{1}$$

The individual terms of the Hamiltonian could be described as follows: The first term of this noninteracting Hamiltonian is the nearest neighbor hopping contribution given by

$$H_0 = -t \sum_{\langle ij\rangle \alpha} c^{\dagger}_{i\alpha} c_{j\alpha}, \tag{2}$$

Where the sum is taken over all pairs of the nearest-neighboring sites, and the operator $c^{\dagger}_{j\alpha}(c_{j\alpha})$ creates (annihilates) an electron with spin α at site j and t is the nearest neighbor hopping amplitude. The values of these parameters for different materials are given in table 1. By performing a Fourier transformation, the first term of the Hamiltonian on the basis of $\{\Psi_{A\uparrow}, \Psi_{A\downarrow}, \Psi_{B\uparrow}, \Psi_{B\downarrow}\}$ reads

$$H_0 = -t \int d^2k \hat{\Psi}^{\dagger}(k) M^0_{4\times 4} \hat{\Psi}(k), \tag{3}$$

where

$$M^0_{4\times4} = \begin{pmatrix} 0 & 0 & \gamma_k & 0 \\ 0 & 0 & 0 & \gamma_k \\ \gamma^*_k & 0 & 0 & 0 \\ 0 & \gamma^*_k & 0 & 0 \end{pmatrix} \tag{4}$$

and $|\gamma_k|^2 = 1 + 4\cos\left(\sqrt{3}/2\ ak_y\right)\cos\left(3/2\ ak_x\right) + 4\cos^2\left(\sqrt{3}/2\ ak_y\right)$. A new type of the SOC arises due to intrinsic buckled geometry of silicene and germanene. In the tight-binding approximation of these graphene-like structures, buckled configuration results in intrinsic Rashba type interaction [1–4]. Hamiltonian of the germanene, which is also a honeycomb structure of group IV elements, is similar to that of the silicene and the difference is just due to the values of various parameter that were listed in table 1. The next term of the Hamiltonian describes the spin-orbit interaction and one has to distinguish between the parallel (with the plane) and perpendicular components of the SOC. The parallel term is as follows

$$H_{SO} = it_{SO} \sum_{\langle\langle ij\rangle\rangle \alpha\beta} u_{ij} c^{\dagger}_{i\alpha} \sigma^z_{\alpha\beta} c_{j\beta}, \tag{5}$$

where t_{SO} is the next-nearest neighbor hopping, $u_{ij} = \frac{\vec{d}_i \times \vec{d}_j}{|\vec{d}_i \times \vec{d}_j|}$ where $\vec{d}_i$ and $\vec{d}_j$ are the two nearest bonds that connect the next-nearest neighbors. Where $u_{ij} = 1$ if the next-nearest neighbor hopping is counterclockwise and $u_{ij} = -1$ when it is clockwise with respect to the positive z axis [5]. The $\langle\langle ij\rangle\rangle$ run over all the next-nearest neighbor hopping sites and σ_z is the Pauli matrix. Performing the Fourier transformation on H_{SO} yields

$$H_{SO} = t_{SO} \int d^2k \hat{\Psi}^{\dagger}(k) M^{SO}_{4\times4} \hat{\Psi}(k), \tag{6}$$

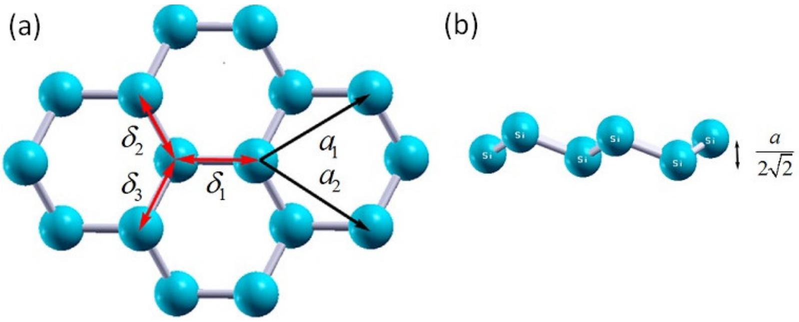

FIG. 1: (Color online) (a) hexagonal structure of buckled two dimensional lattice where $\boldsymbol{\delta}_1 = a\left(1, 0, 1/2\sqrt{2}\right), \boldsymbol{\delta}_2 = a\left(-1/2, \sqrt{3}/2, 1/2\sqrt{2}\right), \boldsymbol{\delta}_3 = a\left(-1/2, -\sqrt{3}/2, 1/2\sqrt{2}\right)$ are the next nearest neighbors position vectors, the lattice vectors are $\boldsymbol{a}_1 = \frac{a}{2}\left(3, \sqrt{3}\right), \boldsymbol{a}_2 = \frac{a}{2}\left(3, -\sqrt{3}\right)$, and the next nearest position vectors are $\boldsymbol{\delta}'_1 = \pm\boldsymbol{a}_1, \boldsymbol{\delta}'_2 = \pm\boldsymbol{a}_2$ and $\boldsymbol{\delta}'_3 = \pm(\boldsymbol{a}_2 - \boldsymbol{a}_1)$. (b) Side view of buckled structure for silicene.

where

$$M^{SO}_{4\times4} = \begin{pmatrix} \eta_k & 0 & 0 & 0 \\ 0 & -\eta_k & 0 & 0 \\ 0 & 0 & -\eta_k & 0 \\ 0 & 0 & 0 & \eta_k \end{pmatrix} \tag{7}$$

and

$$\eta_k = 2\sin\left(k_y a\right) - 4\cos\left(\frac{\sqrt{3}}{2}k_x a\right)\sin\left(\frac{k_y a}{2}\right). \tag{8}$$

The perpendicular term or intrinsic Rashba SOC is given by

$$H_{\text{int}\,R} = -it_{intR}\sum_{\langle\langle ij\rangle\rangle\alpha\beta}\mu_{ij}\hat{c}^{\dagger}_{i\alpha}\left(\vec{\sigma}\times\vec{d}_{ij}\right)^{z}_{\alpha\beta}\hat{c}_{j\beta}, \tag{9}$$

where $t_{\text{int}\,R}$ is the strength of intrinsic Rashba SOC and $\mu_{ij} = \pm1$ for the A (B) site. We can rewrite this term as

$$H_{\text{int}\,R} = -t_{\text{int}\,R}\int d^2k\hat{\Psi}^{\dagger}(k)M^{\text{int}\,R}_{4\times4}\hat{\Psi}(k), \tag{10}$$

in which

$$M^{\text{int}\,R}_{4\times4} = \begin{pmatrix} 0 & 0 & 0 & i\lambda^{+}_{k} \\ 0 & 0 & i\lambda^{-}_{k} & 0 \\ 0 & -i\lambda^{-*}_{k} & 0 & 0 \\ -i\lambda^{+*}_{k} & 0 & 0 & 0 \end{pmatrix}, \tag{11}$$

where $\lambda^{+}_{k} = \lambda^{(1)}_{k} + \lambda^{(2)}_{k}$ and $\lambda^{-}_{k} = \lambda^{(1)}_{k} - \lambda^{(2)}_{k}$,

$$\lambda^{(1)}_{k} = 2i\left[\sin\left(k_y a\right) - \sin\left(\frac{k_y a}{2}\right)\cos\left(\frac{\sqrt{3}}{2}k_x a\right)\right] \tag{12}$$

$$\lambda^{(2)}_{k} = \left[2\sqrt{3}\cos\left(\frac{k_y a}{2}\right)\cos\left(\frac{\sqrt{3}}{2}k_x a\right)\right]. \tag{13}$$

The external Rashba interaction is,

$$H_{extR} = it_{extR}\sum_{\langle ij\rangle\alpha\beta}\hat{c}^{\dagger}_{i\alpha}\left(\vec{\sigma}\times\vec{d}_{ij}\right)^{z}_{\alpha\beta}\hat{c}_{j\beta} \tag{14}$$

where t_{extR} is the strength of extrinsic Rashba SOC. The strength of the external Rashba coupling can be manipulated by an external gate voltage or by the selected substrate. The extrinsic Rashba coupling arises as a result of the inversion symmetry breaking due to an applied perpendicular electric field or interaction with substrate [6]. Similarly this term can be written as

$$H_{extR} = t_{extR} \int d^2k \hat{\Psi}^\dagger(k) M^{extR}_{4\times4} \hat{\Psi}(k), \tag{15}$$

where we have defined

$$M^{extR}_{4\times4} = \begin{pmatrix} 0 & 0 & 0 & i\beta_k^+ \\ 0 & 0 & i\beta_k^- & 0 \\ 0 & -i\beta_k^{-*} & 0 & 0 \\ -i\beta_k^{+*} & 0 & 0 & 0 \end{pmatrix}, \tag{16}$$

where $\beta_k^+ = \beta_k^{(1)} + \beta_k^{(2)}$ and $\beta_k^- = \beta_k^{(1)} - \beta_k^{(2)}$,

$$\beta_k^{(1)} = \exp\left(-i\frac{ak_x}{2\sqrt{3}}\right)\sin\left(\frac{ak_y}{2}\right) \tag{17}$$

$$\beta_k^{(2)} = \frac{\sqrt{3}}{3}\left(\exp\left(i\frac{ak_x}{\sqrt{3}}\right) + \exp\left(-i\frac{ak_x}{\sqrt{3}}\right)\cos\left(\frac{ak_y}{2}\right)\right). \tag{18}$$

The last term is the staggered sub-lattice potential,

$$H_{sub} = l \sum_{i\alpha} \zeta_i E_z^i \hat{c}^\dagger_{i\alpha} \hat{c}_{i\alpha} \tag{19}$$

where l is the distance between the two sub-lattice planes. $\zeta = +1(-1)$ for the A (B) site and E_z is applied electric field perpendicular to the plane. This term can be written as,

$$H_{sub} = l \int d^2k \hat{\Psi}^\dagger(k) M^{sub}_{4\times4} \hat{\Psi}(k), \tag{20}$$

where we have defined,

$$M^{sub}_{4\times4} = \begin{pmatrix} lE_z & 0 & 0 & 0 \\ 0 & lE_z & 0 & 0 \\ 0 & 0 & -lE_z & 0 \\ 0 & 0 & 0 & -lE_z \end{pmatrix}. \tag{21}$$

We have obtained the Hamiltonian throughout the Brillouin zone of buckled honeycomb lattice such as silicene and germanene and also for graphene monolayer but the intrinsic Rashba interaction and last term vanishes for graphene. This is due to the fact that the monolayer graphene has been assumed buckling free.

In general the full Hamiltonian of the system is,

$$H = \begin{pmatrix} \eta_k + lE_z & 0 & \gamma_k & i(\lambda_k^+ + \beta_k^+) \\ 0 & -\eta_k + lE_z & i(\lambda_k^- + \beta_k^-) & \gamma_k \\ \gamma_k^* & -i(\lambda_k^{-*} + \beta_k^{-*}) & -\eta_k - lE_z & 0 \\ -i(\lambda_k^{+*} + \beta_k^{+*}) & \gamma_k^* & 0 & \eta_k - lE_z \end{pmatrix} \tag{22}$$

NUMERICAL DETAILS

Calculation of the wave number dependent quantities should be performed with some care, since the direction of the transferred momentum $\vec{q}$, determines the contribution of each Dirac point. The impurity potential is the Coulomb coupling. The system has been assumed at room temperature where $K_BT = 0.0256$eV. The polarization function has been obtained beyond the Dirac point approximation numerically. Accordingly, a suitable k-space discretization should be chosen. Increasing the density of mesh points should be performed to obtain a proper convergence in obtained physical quantities. In this way we have

chosen different sets of mesh points in the First Brillouin zone. Good convergence has been obtained in the Friedel oscillations by 3702 mesh points which have homogeneously been chosen inside the First Brillouin zone. Meanwhile, most of the numeric calculations have been performed over 13734 mesh points to obtain high accurate results.

Exact diagonalization of the Hamiltonian has been carried out over the selected mesh points. Accordingly, eigenvalues and eigenstates of the k-space Hamiltonian are available numerically on each mesh point. Provided that the eigenstates is given form-factor of the transitions can be calculated. Using the relation

$$\Pi\left(\omega,\vec{q}\right) = \sum_{ss'k} \frac{f_k^s - f_{k+q}^{s'}}{\omega + E_k^s - E_{k+q}^{s'}} F_{s's}\left(\vec{k}+\vec{q},\vec{k}\right), \tag{23}$$

polarization function can be obtained by numerical integration. Then, one can obtain both dielectric function and Friedel oscillations once the polarization function is available.

[1] M. Ezawa, Phys. Rev. Lett. **109**, 055502 (2012).
[2] M. Ezawa, New Journal of Physics **14**, 033003 (2012).
[3] C.-C. Liu, W. Feng, and Y. Yao, Phys. Rev. Lett. **107**, 076802 (2011).
[4] C.-C. Liu, H. Jiang, and Y. Yao, Phys. Rev. B **84**, 195430 (2011).
[5] M. Ezawa, Phys. Rev. B **87**, 155415 (2013).
[6] Y. A. Bychkov and E. I. Rashba, Journal of physics C: Solid state physics **17**, 6039 (1984).
